# Centrosomal Protein 70 Is a Mediator of Paclitaxel Sensitivity

**DOI:** 10.3390/ijms18061267

**Published:** 2017-06-20

**Authors:** Xingjuan Shi, Yujue Wang, Xiaoou Sun, Chan Wang, Peng Jiang, Yu Zhang, Qinghai Huang, Xiangdong Liu, Dengwen Li, Jun Zhou, Min Liu

**Affiliations:** 1 Key Laboratory of Developmental Genes and Human Disease, Institute of Life Sciences, Southeast University, Nanjing 210096, China; wangyujue925@163.com (Y.W.); wangchan6362@126.com (C.W.); jangpeng@163.com (P.J.); 103007392@seu.edu.cn (Y.Z.); hqh@seu.edu.cn (Q.H.); xiangdongliu@seu.edu.cn (X.L.); 2 School of Bioscience and Bioengineering, South China University of Technology, Guangzhou 510006, China; bixosun@scut.edu.cn; 3 State Key Laboratory of Medicinal Chemical Biology, College of Life Sciences, Nankai University, Tianjin 300071, China; dwli@nankai.edu.cn (D.L.); junzhou@nankai.edu.cn (J.Z.); 4 Shandong Provincial Key Laboratory of Animal Resistance Biology, Institute of Biomedical Sciences, College of Life Sciences, Shandong Normal University, Jinan 250014, China

**Keywords:** Cep70, microtubule, paclitaxel sensitivity, breast cancer

## Abstract

Centrosome aberrations have been implicated in the development and progression of breast cancer. Our previous worked show that centrosomal protein 70 (Cep70) regulates breast cancer growth and metastasis. However, it remains elusive whether Cep70 is implicated in the sensitivity of the anti-microtubule drug paclitaxel in breast cancer. Here we provide evidence that Cep70 is a mediator of paclitaxel sensitivity in breast cancer. Cell proliferation assays show that Cep70 expression correlates with paclitaxel sensitivity in breast cancer cell lines. In addition, paclitaxel sensitivity varies when altering Cep70 expression level. Mechanistic studies reveal that Cep70 interacts with tubulin, and promotes the ability of paclitaxel to stimulate microtubule assembly. These data demonstrate that Cep70 mediates paclitaxel sensitivity in breast cancer.

## 1. Introduction

Breast cancer is the most frequently diagnosed cancer and the leading cause of cancer death in women worldwide [1]. With the development of various therapeutic methods, including surgical resection, radiotherapy, and chemotherapy, the outcomes in breast cancer patients have improved in recent decades [2,3]. Paclitaxel, one of the most effective chemotherapy drugs that stabilizes microtubules, has been widely used to treat various cancers [4,5]. However, its effectiveness has been seriously limited by the acquired resistance or variable sensitivity of cancer cells [6]. No predictive marker exists due to the limited knowledge about the mechanism governing paclitaxel sensitivity. There is an urgent need to explore the mechanism underlying the modulation of paclitaxel sensitivity, which will help to improve response rates and potentially extend survival in these patients.

The centrosome plays an important role in mitosis by organizing the microtubule network and coordinating bipolar spindle formation [7]. Centrosome abnormalities have been found in most human cancers, and have been shown to be essential in the initiation and development of cancers [8,9]. The correlation between expression of centrosome proteins and sensitivity to chemotherapeutic agents, which might be a potential target for cancer therapy, has attracted increasing attention [10,11,12,13]. It is shown that centrosomal protein 70 (Cep70) plays a critical role in the development and progression of breast cancer [14]. Moreover, it is suggested that the restricted and localized position of Cep70 makes it a potential therapeutic target [15]. However, the role Cep70 plays in paclitaxel sensitivity of cancer cells remains unknown. In this study, we provide the first evidence that Cep70 modulates paclitaxel sensitivity by modulating the effect of paclitaxel on microtuble assembly.

## 2. Results

### 2.1. Centrosomal Protein 70 (Cep70) Expression and Paclitaxel Sensitivity in Breast Cancer Cells

**Figure 1:**
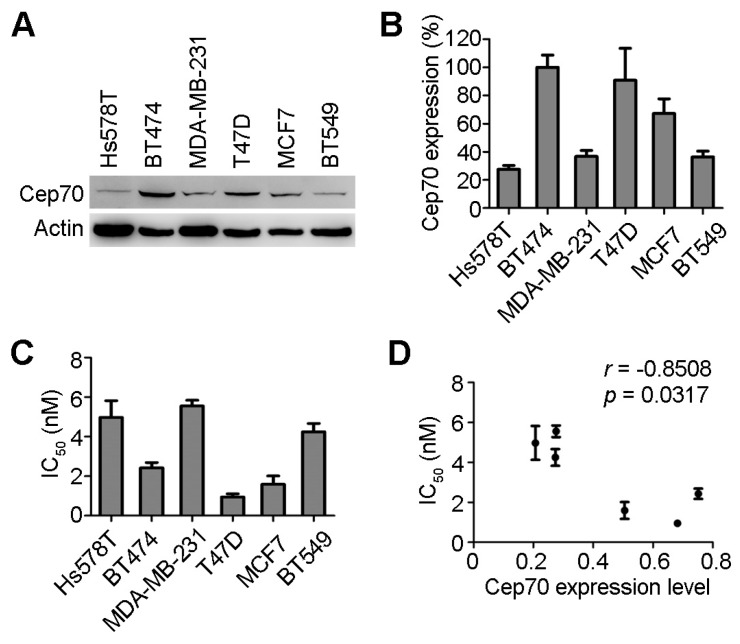
Centrosomal protein 70 (Cep70) expression and paclitaxel sensitivity in breast cancer cells. (**A**) The protein expression levels of Cep70 and actin in human breast cancer cells were examined by immunoblot analysis; (**B**) Experiments were performed as in (**A**), and the level of Cep70 expression in breast cancer cell lines was quantified by measuring the intensity of immunoblot band with Image J software (National Institutes of Health). Actin level was used as an internal control. The value of *y*-axis showed Cep70 expression level in different cancer cells as a percentage of that in BT474 cell; (**C**) The breast cancer cells were treated with paclitaxel for 48 h, and the IC_50_ values were then examined by cell proliferation assay; (**D**) Experiments were performed as in (**C**), and the relationship between IC_50_ and the expression level of Cep70 was analyzed by Spearman rank correlation test. All data are presented as means ± standard deviations (SD), *n* = 3 per group.

We detected the protein level of Cep70 in six breast cancer cell lines (Hs578T, BT474, MDA-MB-231, T47D, MCF7, BT549) by immunoblotting, and found that the expression of Cep70 is different in these cell lines (Figure 1A). BT474, T47D, and MCF7 cells had much higher Cep70 expression than the other cell lines (Figure 1B). The IC_50_ values of paclitaxel, the concentration of paclitaxel in preventing cell proliferation by 50%, were used to measure the sensitivity to paclitaxel of cancer cells. Cell proliferation assays were performed with cells administered with different concentrations of paclitaxel. We found that BT474, T47D, and MCF7 cells with relatively higher Cep70 expression tended to have much lower IC_50_ values than the other cell lines (Figure 1C).

To further study the correlation between Cep70 expression and sensitivity to paclitaxel in breast cancer cells, the relationship between IC_50_ and the relative Cep70 expression levels was analyzed. As shown in Figure 1D, the expression level of Cep70 negatively correlated with the IC_50_ values of paclitaxel in breast cancer cells (*r* = −0.851, *p* = 0.031). Although paclitaxel sensitivity can vary between cancer lines for reasons not fully understood, these results suggest that Cep70 expression level may be a factor in the sensitivity to paclitaxel in breast cancer cells.

### 2.2. Cep70 Overexpression Enhances the Sensitivity of Breast Cancer Cells to Paclitaxel

**Figure 2:**
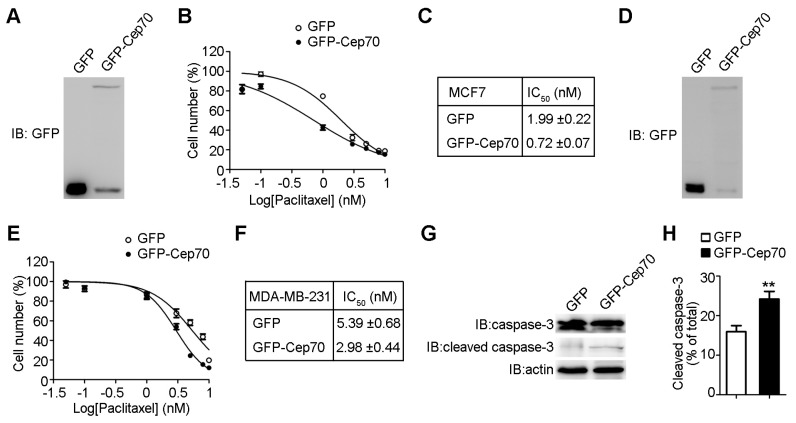
Cep70 overexpression enhances the sensitivity of breast cancer cells to paclitaxel. (**A**) MCF7 cells were transfected with GFP (Green fluorescent protein) or GFP-Cep70, and then collected for immunoblot analysis with anti-GFP antibody; (**B**) MCF7 cells transfected with GFP or GFP-Cep70 were treated with paclitaxel for 48 h and the number of cells determined by cell proliferation assay; (**C**) Experiments were performed as in (**B**) and IC_50_ values were analyzed; (**D**) MDA-MB-231 cells transfected with GFP or GFP-Cep70 were collected, and examined by immunoblot analysis; (**E**) MDA-MB-231 cells transfected with GFP or GFP-Cep70 were incubated with paclitaxel for 48 h, and the number of cells was determined; (**F**) Experiments were performed as in (**E**) and IC_50_ values were measured; (**G**) MCF7 cells transfected with GFP or GFP-Cep70 were treated with 2 nM paclitaxel for 48 h, and activation of caspase-3 was then analyzed by immunoblot analysis; (**H**) Experiments were performed as in (**G**), and the level of caspase-3 activation was determined as the amount of cleaved caspase-3 as a percentage of the total caspase. All data are presented as means ± standard deviations (SD), *n* = 3 per group. ** *p* < 0.01.

To study the role of Cep70 in paclitaxel sensitivity, we first increased Cep70 expression level in MCF7 cells by transfecting GFP (Green fluorescent protein) or GFP-Cep70 (Figure 2A). We found that overexpression of Cep70 leads to higher paclitaxel sensitivity in MCF7 cells (Figure 2B). In the control group, cells showed an IC_50_ of 1.99 nM, however the IC_50_ of cells with Cep70 overexpression was 0.72 nM (Figure 2C). To further determine the role Cep70 plays in paclitaxel sensitivity modulation, we transfected MDA-MB-231 cells with GFP-Cep70 or GFP (Figure 2D). The overexpression of Cep70 also rendered MDA-MB-231 cells more sensitive to the paclitaxel (Figure 2E). The IC_50_ value in MDA-MB-231 cells transfected with GFP was 5.39 nM, which decreased to 2.98 nM in cells with Cep70 overexpression (Figure 2F). Paclitaxel-treated cells are known to undergo apoptosis, we thus checked whether Cep70 affects the activity of paclitaxel to trigger apoptosis in breast cancer cells. We found that overexpression of Cep70 increases the level of cleaved caspase-3, indicating that Cep70 enhances the ability of paclitaxel to trigger apoptosis (Figure 2G,H).

### 2.3. Knockdown of Cep70 Expression Reduces the Sensitivity to Paclitaxel of Breast Cancer Cells

**Figure 3:**
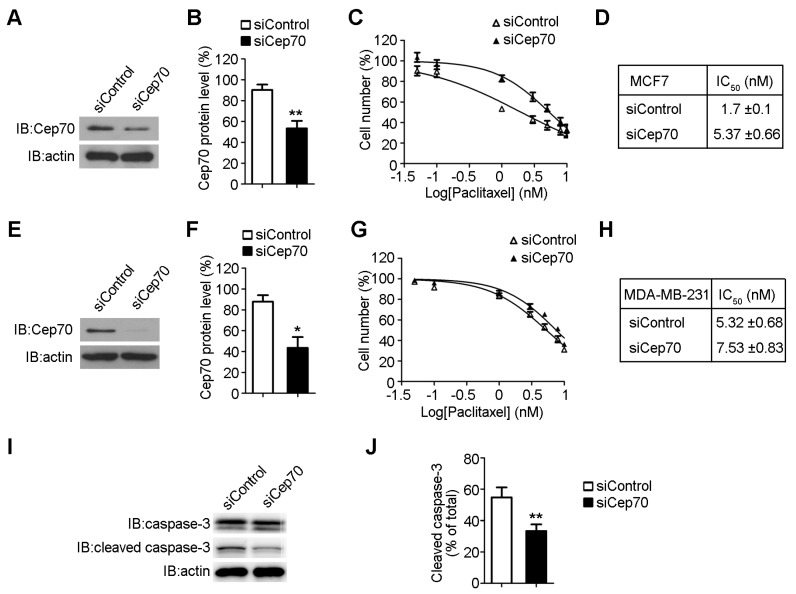
Knockdown of Cep70 expression reduces the sensitivity to paclitaxel of breast cancer cells. (**A**) MCF7 cells were transfected with control or Cep70 siRNA, and then collected for immunoblot analysis; (**B**) Experiments were performed as in (**A**), and the protein level of Cep70 was analyzed; (**C**) MCF7 cells transfected with control or Cep70 siRNA were treated with paclitaxel for 48 h. Cell proliferation was examined; (**D**) Experiments were performed as in (**C**) and the IC_50_ was analyzed; (**E**) MDA-MB-231 cells were transfected with control or Cep70 siRNAs, and then collected for immunoblot analysis; (**F**) Experiments were performed as in (**E**), and the protein level of Cep70 was analyzed; (**G**) MDA-MB-231 cells were transfected with control or Cep70 siRNA, and then treated with various concentrations of paclitaxel for 48 h. Cell proliferation was examined; (**H**) Experiments were performed as in (**G**) and the IC_50_ was calculated; (**I**) MCF7 cells transfected with control or Cep70 siRNA were treated with 2 nM paclitaxel for 48 h, and activation of caspase-3 was analyzed by immunoblot analysis; (**J**) Experiments were performed as in (**I**), and the level of caspase-3 activation was determined as the amount of cleaved caspase-3 as a percentage of the total caspase. All data are presented as means ± standard deviations (SD), *n* = 3 per group. * *p* < 0.05; ** *p* < 0.01.

To further verify the function of Cep70 in paclitaxel sensitivity, we reduced Cep70 expression level in breast cancer cells. Immunoblot analysis showed that Cep70 siRNAs significantly decreased Cep70 expression level in both MCF7 cells (Figure 3A,B) and MDA-MB-231 cells (Figure 3E,F). Paclitaxel IC_50_ of MCF7 cells transfected with control siRNA was 1.7 nM, which increased to 5.37 nM in cells transfected with Cep70 siRNA (Figure 3C,D). In addition, depletion of Cep70 expression elevated the IC_50_ value of paclitaxel from 5.32 to 7.53 nM in MDA-MB-231 cells (Figure 3G,H). We further examined caspase-3 activation of cells with decreased Cep70 expression, and found that knockdown of Cep70 reduces the effect of paclitaxel on caspase-3 activation (Figure 3I,J).

### 2.4. Cep70 Promotes the Capability of Paclitaxel to Induce Microtubule Assembly

**Figure 4:**
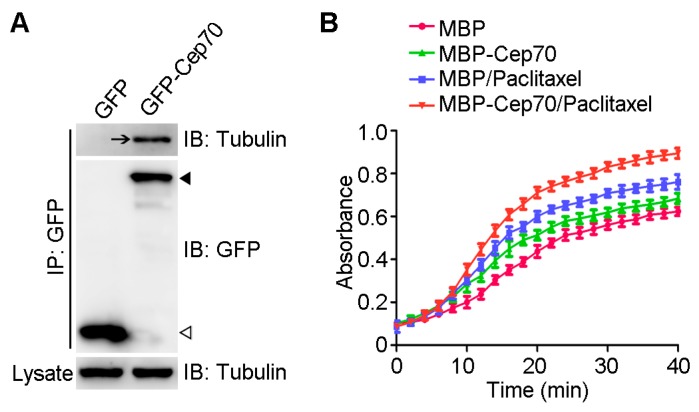
Cep70 promotes the capability of paclitaxel to induce microtubule assembly. (**A**) GFP-Cep70 or GFP was transfected into 293T cells, and immunoprecipitation was then carried out with anti-GFP antibody-conjugated agarose beads. Cell lysates and the immunoprecipitates were examined by immuoblot analysis with anti-tubulin or anti-GFP antibodies. IP represents immunoprecipitate, and IB represents immunoblot analysis. Arrow shows pull-down of tubulin. Black arrowhead and hollow arrowhead indicates immunoprecipitate of GFP-Cep70 and GFP, respectively; (**B**) MBP (Maltose binding protein) or MBP-Cep70 (10 μM) was incubated with tubulin (10 μM) at 37 °C before adding paclitaxel (10 μM), and the microtubule polymerization was detected by recording the optical absorbance at 350 nm. All data are presented as means ± standard deviations (SD), *n* = 3 per group.

Paclitaxel is an anticancer drug that promotes microtubule assembly by suppressing its dynamic instability [16]. To gain more insights into the role of Cep70 in modulating paclitaxel sensitivity, we first examined whether Cep70 interacts with tubulin. Immunoprecipitation and pull-down assays showed that tubulin is present in the pull-down preparation of GFP-Cep70 (Figure 4A), showing that Cep70 interacts with tubulin. To study the role of Cep70 on paclitaxel-mediated microtubule assembly, we purified MBP (maltose binding protein) and MBP-Cep70 from bacteria, and examined their impact on microtubule assembly in vitro. By measuring the changes in optical absorbance at 350 nm wavelength, we found that Cep70 enhanced the capability of paclitaxel to promote microtubule assembly (Figure 4B).

## 3. Discussion

The centrosome acts as microtubule organizing center and plays a crucial role in coordinating bipolar spindle formation during mitosis [7]. It is reported that centrosome abnormalities result in dyspoiesis of the mitotic spindle, which blocks the therapeutic targets of spindle poisons, such as paclitaxel [10,11,12,13]. Paclitaxel is a microtubule polymerizing agent that blocks mitotic progression by activating the spindle assembly checkpoint [17]. Recent studies have shown that Cep70 regulates mitotic spindle organization and participates in the development and progression of breast cancer [18]. It has also been suggested that the role Cep70 plays in cancer might be a consequence of the interaction of Cep70 with other microtubule associated proteins [15]. Therefore, we hypothesized that the abnormal expression of Cep70 might influence the sensitivity of breast cancer cells to paclitaxel. Herein, we provide the first evidence that Cep70 modulates the sensitivity of breast cancer cells to paclitaxel.

In this study, we provide several lines of evidence showing a novel role of Cep70 in modulating sensitivity to paclitaxel of cancer cells: Cep70 expression level is related to paclitaxel sensitivity in breast cancer cell lines; Cep70 overexpression increases the paclitaxel sensitivity of breast cancer cells while knockdown of Cep70 shows the opposite effect. It will be worthwhile to investigate whether the expression level of Cep70 in breast cancer tissues correlates with the pathological response of tumors to paclitaxel-based chemotherapy. Elucidation of this will help provide novel insights into the predictive markers of the response to paclitaxel-based chemotherapy.

It has been reported that Cep70 participates in the development and progression of breast cancer [14]. The present study investigated the potential mechanism of how Cep70 modulates paclitaxel sensitivity of breast cancer cells. We demonstrate that Cep70 enhances the ability of paclitaxel to induce apoptosis in breast cancer cells. In addition to our previous finding that Cep70 binds to centrosome marker γ-tubulin, we have also shown here for the first time that Cep70 interacts with tubulin in cells. This finding makes sense because Cep70 is localized to the centrosome and distributes in the cytoplasm as well [18]. Moreover, by the light scattering assay, we found that Cep70 promotes the ability of paclitaxel to induce microtubule assembly. Given our previous finding that Cep70 regulates microtubule stability [19], we speculate that Cep70 might modulate paclitaxel sensitivity in breast cancer cells by enhancing the ability of paclitaxel to stimulate microtubule assembly and stabilization, and then cause mitotic arrest and apoptosis.

At present, it remains unknown how Cep70 increases the capability of paclitaxel to induce microtubule assembly. It is likely that Cep70 promotes the interaction of paclitaxel with the microtubule by structural or allosteric effects, which is similar to some microtubule associated proteins, such as CLIP-170 [20,21]. Given the role of Cep70 in microtuble organization, it is also conceivable that Cep70 increases the microtubule stabilizing ability of paclitaxel by promoting microtubule elongation [19,22,23,24]. It will be interesting to explore other potential mechanisms underlying the modulation of paclitaxel sensitivity by Cep70, which might help to improve response rates and provide theoretical evidence for clinical treatment of breast cancer.

## 4. Materials and Methods

### 4.1. Materials

Paclitaxel, Sulforhodamine B (SRB), antibodies against tubulin and β-actin were purchased from Sigma-Aldrich (St. Louis, MO, USA). Antibodies against GFP and caspase-3 were purchased from Roche (Amherst, CA, USA) and Cell Signaling (Beverly, MA, USA), respectively. Antibody against Cep70 was generated as described previously [18]. Antibody dilution of 1:1000 for all the primary antibodies was used in the study. Human Cep70 siRNAs (5′-GAGGAUGAAUCACUAAGUA-3′) and luciferase control siRNA (5′-CGUACGCGGAAUACUUCGA-3′) were synthesized by Ribobio (Guangzhou, China).

### 4.2. Plasmids and Proteins

Expression plasmids for GFP-Cep70 and MBP-Cep70 were generated by using the pEGFPC1 and pMALp2T vectors, respectively. MBP (maltose binding protein) or MBP-Cep70 fusion proteins were purified with amylose resin following the manufacturer’s protocol (New England Biolabs, Ipswitch, MA, USA). MAP-free tubulin was purchased from Cytoskeleton (Denver, CO, USA).

### 4.3. Cell Culture and Transfection

All human breast cancer cells were purchased from the American Type Culture Collection (Manassas, VA, USA). HS578T, BT474, and MCF7 cells were cultured in DMEM (Dulbecco’s modified Eagle’s medium) medium. MDA-MB-231, T47D, and BT549 cells were cultured in the RPMI-1640 medium. All cell lines were maintained at 37 °C in a 5% CO_2_ humidified atmosphere. Lipofectamine 3000 and Lipofectamine RNAiMAX reagents (Thermo Fisher Scientific, Carlsbad, CA, USA) were used for plasmids and siRNAs transfection into cells, respectively. Cells cultured in 6-well plates were transfected with plasmids (6 μg) or siRNA (90 pmol) for 12 h, and then treated with paclitaxel.

### 4.4. Immunoblot Analysis

Cells were collected after transfection and lysed in RIPA lysis buffer (Beyotime, Shanghai, China). Protein lysates were separated by SDS-PAGE (Sodium dodecyl sulfate-Polyacrylamide gel electrophoresis) and transferred onto polyvinylidene difluoride membranes (Millipore, Billerica, MA, USA). Membranes were blocked in 5% fat-free milk dissolved in TBST (Tris Buffered Saline with Tween) (10 mM Tris, pH 8.0, 150 mM NaCl, 0.5% Tween) for 2 h and incubated with primary antibodies overnight at 4 °C. After exposure to the secondary horseradish peroxidase-conjugated secondary antibodies (Santa Cruz Biotechnology, Dallas, TX, USA, 1:5000 dilution) for 1 h at room temperature, proteins were examined with enhanced chemiluminescence detection reagent (Millipore).

### 4.5. Cell Proliferation Assay

The impact of paclitaxel on cell viability was examined by SRB staining as described before [25]. Cells were cultured in 96-well plates and treated with different concentrations of paclitaxel for 48 h. The medium was discarded after treatment, and cold trichloroacetic acid (10% *w*/*v*) was added to each well to fix the cells. The plates were then washed with deionized water and dried in the air. Cells were stained with sulforhodamine B (0.4% *w*/*v* in 1% acetic acid), and then washed with 1% acetic acid. Tris base (10 mM) was used to extract the dye, and the absorbance was measured at 562 nm. IC_50_, the concentration of drug that prevents cell proliferation by 50% was identified. For each experiment, data are given from three independent experiments performed in technical triplicates.

### 4.6. Immunoprecipitation

Cell lysates were incubated with anti-GFP antibody-conjugated agarose beads (MBL) at 4 °C overnight. The beads were washed with RIPA lysis buffer thoroughly and denatured in SDS loading buffer. The samples were separated by SDS–PAGE and examined by immunoblot analysis.

### 4.7. In Vitro Tubulin Polymerization Assay

Tubulin polymerization buffer consisted of 100 mM PIPES, 1 mM EGTA, 1 mM MgSO_4_, and 1 mM GTP (pH 6.8). Spectrophotometer cuvettes were kept at room temperature before adding paclitaxel, tubulin, and MBP-Cep70 or MBP, and then transferred to a spectrophotometer (Amersham Biosciences, Switzerland) at 37 °C. Tubulin polymerization into microtubules was examined by measuring the absorbance at 350 nm wavelength as described before [26]. Experiments were performed in triplicate and repeated at least three times.

### 4.8. Statistics Analysis

Results are expressed as the mean ± S.D. Statistics analysis was performed using the Student’s *t*-test. Correlation coefficient was calculated by the Spearman’s rank correlation test.

## 5. Conclusions

Our findings demonstrate that Cep70 modulates the sensitivity of breast cancer cells to paclitaxel. We found that Cep70 expression level is related to paclitaxel sensitivity in breast cancer cell lines. Mechanistic studies showed that Cep70 enhances the ability of paclitaxel to induce apoptosis in breast cancer cells. In addition, the interaction of Cep70 with tubulin indicated that Cep70 might modulate paclitaxel sensitivity by enhancing the ability of paclitaxel to stimulate microtubule assembly, and then cause mitotic arrest and apoptosis. The study will help provide insights into the predictive markers of the response to paclitaxel-based chemotherapy.
